# Malonyl/Acetyltransferase (MAT) Knockout Decreases Triacylglycerol and Medium-Chain Fatty Acid Contents in Goat Mammary Epithelial Cells

**DOI:** 10.3390/foods11091291

**Published:** 2022-04-29

**Authors:** Weiwei Yao, Jun Luo, Huibin Tian, Huimin Niu, Xuetong An, Xinpei Wang, Saige Zang

**Affiliations:** Shaanxi Key Laboratory of Molecular Biology for Agriculture, College of Animal Science and Technology, Northwest A&F University, Yangling, Xianyang 712100, China; yaoweiwei@nwafu.edu.cn (W.Y.); tianhuibin@nwafu.edu.cn (H.T.); niuniu0866@foxmail.com (H.N.); anxuetong996@163.com (X.A.); wxp1846@163.com (X.W.); zsg0304@126.com (S.Z.)

**Keywords:** *MAT*, *FASN*, CRISPR/Cas9, acetyl-CoA

## Abstract

Malonyl/acetyltransferase (MAT) is a crucial functional domain of fatty acid synthase (FASN), which plays a vital role in the de novo synthesis of fatty acids in vivo. Milk fatty acids are secreted by mammary epithelial cells. Mammary epithelial cells are the units of mammary gland development and function, and it is a common model for the study of mammary gland tissue development and lactation. This study aimed to investigate the effects of MAT deletion on the synthesis of triacylglycerol and medium-chain fatty acids. The MAT domain was knocked out by CRISPR/Cas9 in the goat mammary epithelial cells (GMECs), and in MAT knockout GMECs, the mRNA level of FASN was decreased by approximately 91.19% and the protein level decreased by 51.83%. The results showed that MAT deletion downregulated the contents of triacylglycerol and medium-chain fatty acids (*p* < 0.05) and increased the content of acetyl-Coenzyme A (acetyl-CoA) (*p* < 0.001). Explicit deletion of *MAT* resulted in significant drop of *FASN*, which resulted in downregulation of *LPL, GPAM, DGAT2, PLIN2, XDH, ATGL, LXRα*, and *PPARγ* genes in GMECs (*p* < 0.05). Meanwhile, mRNA expression levels of *ACC, FASN, DGAT2, SREBP1*, and *LXRα* decreased following treatment with acetyl-CoA (*p* < 0.05). Our data reveals that FASN plays critical roles in the synthesis of medium-chain fatty acids and triacylglycerol in GMECs.

## 1. Introduction

Goat milk is one of the richest foods [1,2]; it is characterized by rich nutrition, good protein quality, minimal allergens, small fat particles, and easy digestion and absorption [3]. Milk fatty acids are high in nutrition [4,5,6,7], shown through evaluation of milk quality [8]. Saturated fatty acid (SFA) is the main fatty acid type in ruminant milk [9,10]. The content of medium-chain fatty acids in goat milk is high, especially C6:0, C8:0, and C10:0 [1,11,12,13]. These medium-chain fatty acids have a positive role in weight loss [14] and cholesterol reduction [15,16], possessing antibacterial properties [17] and treating gastrointestinal diseases [18,19]. This gives goat milk special therapeutic properties in certain aspects of human nutrient metabolism [1,20,21]. However, C12:0, C14:0, and C16:0 increase the levels of low-density lipoprotein (LDL) in the blood, which is detrimental to the health of the body [22,23].

Different fatty acid compositions have diverse functions and meet the needs of various populations [1]. Controlling fatty acid composition remains a worthwhile topic for future research [24]. The composition of fatty acids in milk is responsive to nutritional factors in the diet of goats [25]. Dietary manipulation is a common method of changing the composition of fatty acids in milk [19,26]. However, this method also has some disadvantages, such as low efficiency [19] and high cost [27]. Moreover, dietary control was found to regulate fatty acid composition through affecting fatty acid metabolism gene function [28]. Therefore, regulation of fatty acid composition by fatty acid metabolism genes should be considered.

Compared with plant breeding, gene editing breeding is seldom used in animal husbandry [27]. Large livestock such as goats have high breeding costs, long breeding cycles, and complex breeding processes [27]. Meanwhile, fatty acids in milk are synthesized by mammary epithelial cells [29]. Understanding the effect of key genes on fatty acid composition of mammary epithelial cells is the basis of molecular breeding [7].

The effects of FASN on de novo fatty acid synthesis have been confirmed in humans [30], cattle [27], mice [31], and goats [32]. Several studies reported that the expression level of FASN is related to the fatty acids composition in milk [31,32,33]. Most short- and medium-chain fatty acid synthesis in goat milk comes from the de novo fatty acid synthesis pathway [13,34], and FASN is a key enzyme in de novo synthesis of fatty acids in GMECs [35]. Deletion of FASN in mammary epithelial cells of non-ruminants leads to the decrease in the contents of medium- and long-chain fatty acids and total fatty acids in milk [31]. Inhibition of FASN expression in mammary epithelial cells in ruminants reduces the content of medium-chain fatty acids (C10:0, C12:0, C14:0) in milk [31,36,37]. The overexpression of FASN increases the percentage of C12:0 [21].

The nutrition and flavor could be altered by modifying fatty acid composition of milk. In this study, MAT-knockout goat mammary epithelial cells (GMECs) were generated using CRISPR/Cas9, aiming to investigate the effects of MAT on fatty acid composition. Here, we found that the contents of medium-chain fatty acids and triacylglycerol decreased, and the unsaturated fatty acids increased, in MAT-knockout cells. These data provide a useful way to regulate the composition of milk fatty acids through gene handling.

## 2. Materials and Methods

### 2.1. Ethics Statement

All the experimental procedures were permitted by the Animal Care and Use Committee of the College of Animal Science and Technology in Northwest A&F University, Yang Ling, China (permit number: 15-516).

### 2.2. sgRNA Design and Vector Construction

The goat *FASN* sequence was downloaded from NCBI website (https://www.ncbi.nlm.nih.gov/, accessed on 24 November 2019), the MAT fragment included part of exons from 9 to 15. Then, sgRNA was designed according to CHOPCHOP website (https://zlab.bio/guide-design-resources, accessed on 24 November 2019) [38]. *Bbs*I and protective sequence were added to both ends of the sgRNA sequence, and double-stranded DNA was generated by annealing. Then, the all-in-one vector pSpCas9 (BB)-2A-Puro (plasmid 62988, PX459 V2.0, Addgene, Cambridge, MA, USA), a gift from Feng Zhang [39], was digested by *Bbs*I enzyme (R3539S, NEB, Ipswich, MA, USA) and linked to double-stranded DNA. The connect product was transferred into *E. coli* (CB171211, TIANGEN, Beijing, China) for amplification. Single colonies on the solid medium (tryptone 10 g/L, yeast extract 5 g/L, NaCl 10 g/L, Agaragar 15 g/L) were picked and placed in a liquid LB medium (tryptone 10 g/L, yeast extract 5 g/L, NaCl 10 g/L) for overnight propagation, and the plasmids were extracted for sequencing (Tsingke Biotechnology Co Ltd., Beijing, China). The vector successfully ligated with sgRNA was screened, the PX459-sgRNA plasmid vector was extracted with HighPure Mini Plasmid Kit (DP201101X, TIANGEN, Beijing, China). The plasmid was stored at −20 °C and its concentration and quality were detected by spectrophotometer (Nanodrop 2000, Thermo Fisher Scientific, Rockford, IL, USA) before use.

### 2.3. Isolation of GMECs

Dairy goats during the peak lactation period (60 days after parturition) were anesthetized by xylazine hydrochloride (0.01 mL/kg, Huamu Animal Health Products Corporation, Shandong, China) before surgery [40], then operated according to the previous purification studies [41]. The breast tissues were cut into small pieces and cultured in an incubator at 37 °C and 5% CO_2_, with the medium changed every two days until the mammary epithelial cells were isolated from the breast tissues block. The basal growth medium contained 90% DMEM/F12 medium (SH30023−01, Hyclone, Logan, UT, USA), and 10% fetal bovine serum (10099-141, Invitrogen, Waltham, MA, USA), with 5 mg/L bovine insulin (16634, Sigma, St. Louis, MO, USA), 5 μg/mL hydrocortisone (H0888, Sigma, St. Louis, MO, USA), 100 U/mL penicillin/streptomycin (080092569, Harbin Pharmaceutical Group, Harbin, PR China), and 10 ng/mL epidermal growth factor (PHG0311, Invitrogen, Waltham, MA, USA) added. Then, GMECs were stored in liquid nitrogen and resuscitated before use.

### 2.4. Cells Culture, Transfection, and Screening

The frozen primary mammary epithelial cells were thawed in a 37 °C wet bath, quickly added into a 37 °C preheated culture medium, centrifuged at 1000 rpm for 4 min, and the supernatant was discarded. After being blown evenly with the fresh culture medium, the cells were evenly spread into a 60 mm dish and cultured in a 5% CO_2_ and 37 °C incubator, the fresh culture medium was replaced every day for culture and passage until the cell state was stable. The cells were digested by the ATV (digestive fluid, Trypsin 2.5 g/L, NaCl 8 g/L, KCl 0.4 g/L, D-G 1 g/L, NaHCO_3_ 0.58 g/L, EDTA-Na_2_ g/L) and evenly spread in the six-well plate. When the cells confluency reached approximately 70% to 80%, the cells were transfected with Lipofectamine^TM^ 2000 (11668019, Invitrogen, Waltham, MA, USA) transfection reagent, and the control group was set. The plasmid and Lipofectamine^TM^ 2000 were diluted simultaneously with DMEM/F12 (SH30023−01, Hyclone, Logan, UT, USA). Plasmids and transfection reagents were mixed and incubated for 15 min, then dropped into a six-well plate for gentle mixing. After 48 h of transfection, 1.0 μg/mL puromycin (P8833, Sigma, St. Louis, MO, USA) was added for screening. About 96 h later, the cells death status was examined through a microscope. After all the normal cells died, the normal medium was replaced for continuous culture until cell clusters appeared. All the dead cells except a single round cells cluster was scraped off with a gun head and carefully digested to the new medium.

### 2.5. Cells Culture and T7EN1 Assay

The GMECs were digested, half were used for T7 Endonuclease I (M0302L, NEB, Ipswich, MA, USA) cleavage assay [42], and the other half was cultured continuously. The cells genome was extracted using a Universal Genomic DNA Kit (CW2298S, CW Biotech, Beijing, China). Extraction of genomes from cells with a Universal Genomic DNA Kit (CW2298S, CW Biotech, Beijing, China). Utilizing the genome as the template, the genome fragment of about 500 bp near the sgRNA locus was amplified by PCR using PrimeSTAR Max DNA Polymerase (R045A, Takara Bio Inc., Otsu, Japan), then detected it in a 1% agarose gel. If the band was single, the solution be purified by a PCR Clean-Up Kit (AP-PCR-50, Axygen, Union City, CA, USA) according to the manufacturers instructions. The purified DNA fragments were annealed in NEB buffer 2 (B7202S, NEB, Ipswich, MA, USA), followed by the addition of 0.3 μL T7 Endonuclease I (M0302L, NEB, Ipswich, MA, USA), reaction at 37 °C for 30 min, and results of enzyme digestion were identified by 2% agarose electrophoresis. The selected DNA fragments were ligated into Pmd19-T vector (CB35526014, takara Bio Inc., Otsu, Japan), transferred to *E. coli* (CB171211, TIANGEN, Beijing, China), and monoclonal colonies were selected and sequenced, which were compared with the gene sequence of the control group.

### 2.6. Genotypic Structure Prediction

The DNA sequence obtained by sequencing was translated into an amino acid sequence using BioXM2.6 software (http://nome.njau.edu.cn/biom, accessed on 24 July 2020). The amino acid sequence was input to Protparam online website (https://web.expasy.org/protparam, accessed on 24 July 2020) for protein physical property analysis. Entered NPS@ online (http://npsa-pbil.ibcp.fr, accessed on 24 July 2020) for secondary structure analysis. Entered SWISS-MODEL online website (https://swissmodel.expasy.org/interactive, accessed on 24 July 2020) for tertiary structure prediction.

### 2.7. RNA Extraction and Real-Time Quantitative PCR (RT-qPCR)

Quantitative primers were designed for *MAT* region using AlleleID 6 software (http://www.premierbiosoft.com/, accessed on 25 July 2020), Sense Primer: ATGGCGGCTGTAGGCTTGAC, Anti-Sense Primer: GCACCTCCTTGGCGAACACG. RNAiso Plus (9109, Takara Bio Inc., Otsu, Japan) was added to the cells, and RNA in the cells was extracted according to the manufacturer’s protocol. The RNA was reversely transcribed into cDNA with the PrimeScript RT Reagent Kit (RR820A, Perfect Real Time, Takara Bio Inc., Otsu, Japan), and the mRNA expression levels of *MAT* gene in each group were detected quantitatively with SYBR Premix Ex TaqII (RR820A, Perfect Real Time, Takara Bio Inc., Otsu, Japan) by Light Cycler 96 Real-Time PCR system (Roche Diagnostics Ltd., Mannheim, Germany). Using *UXT* and *GAPDH* as internal reference genes, the relative expression values were calculated by the 2^−ΔΔCt^ method. Each set of tests consisted of three technical and three biological replicates. The primers of target genes are listed in Table S1.

### 2.8. Protein Extraction and Western Blot Assay

The total protein of NC-GMECs and MAT-KO-GMECs were, respectively, extracted and lysed in ice-cold RIPA buffer (R0010, Solarbio, Beijing, China) with protease inhibitor (04693132001, Roche Diagnostics Ltd., Mannheim, Germany). Protein concentration was measured by BCA Protein Assay Kit (23227, Thermo Fisher Scientific, Rockford, IL, USA). The size of FASN protein was about 272 kD, and that of *β*-actin protein was about 42 kD. The amount of protein loading was 20 ng. After SDS-PAGE electrophoresis, the bands were transferred to FVDF membrane (HATF00010, Millipore, Burlington, MA, USA) by semi-dry transfer method. After the transfer membrane was completed, blocking was performed with 5% skim milk (232100, BD, Franklin Lakes, NJ, USA), and antibodies (FASN, bs-1498R; *β*-actin, CW0096) were incubated. The horseradish–peroxidase (HRP)-conjugated goat anti-rabbit-IgG (CW0103, CW Biotech, Beijing, China) and goat anti-mouse-IgG (CW0102) were used as secondary antibodies, and the target band signals were exposed by ECL Western blot system (1705061, Bio-Rad, Hercules, CA, USA).

### 2.9. Off-Target Analysis

The online website Cas-OFFinder (http://www.rgenome.net/cas-offinder/, accessed on 8 August 2020) was utilized to predict the off-target sites [43]. The choices were: SpCas9, 5′-NGG-3′, Capra hircus, and mismatch number (eq or less than) 3 [44]. Ten high-probability target sites were screened, and corresponding sequences were found at genomic positions. Primers (Table S2) were designed (www.PremierBiosoft.com, accessed on 8 August 2020) to amplify the fragment on the genome, and T7EN1 (M0302L, NEB, Ipswich, MA, USA) digestion test was performed.

### 2.10. Measurement of Total Cellular Triacylglycerol

MAT-KO-GMECs and NC-GMECs were spread on a 6-well-plate, and collected after the cells upon reaching 90% confluence. The triacylglycerol in the cells were extracted by the Tissue Triacylglycerol Assay Kit (E1013, Applypen Technologies Inc., Beijing, China). The sample contents were read at 550 nm by a Biotek Microplate Reader (Winooski, VT, USA). The relative content of triacylglycerol was corrected by intracellular protein levels and displayed as micrograms per milligram of protein (μg/mg protein) [45]. Protein content was determined by a BCA Protein Assay Kit (23227, Thermo Fisher Scientific, Rockford, IL, USA).

### 2.11. Measurement of Intracellular Fatty Acid Composition

The MAT-KO-GMECs and NC-GMECs were spread on a 6-well-plate. The samples were methylated with 2.5% (*v*/*v*) methanol sulfate, collected into a glass tube, sonicated for 10 min, and sealed at 80 °C for 1 h. Then, added hydrochloric acid and normal hexane. The supernatant was charged with anhydrous sodium sulfate, shaken, and allowed to stand overnight [46]. The supernatant was centrifuged and placed in a vial for GC analysis by gas chromatography (Agilent 7890A; Agilent Technologies Inc., CA, USA) with 100 m HP-5 column (Agilent Technologies Inc.).

### 2.12. Measurement of Genes Related to Intracellular Fatty Acid Metabolism

Using *UXT* and *GAPDH* as internal reference genes, the expression levels of fatty acid synthesis genes (*ACC, FASN*), triacylglycerol synthesis genes (*GPAM*, *DGAT1, DGAT2, AGPAT6*), lipid droplet secretion genes (*PLIN2, XDH, TIP47*), triacylglycerol hydrolysis genes (*LPL, ATGL*), and regulatory factors (*LXRα, PPARγ, SREBP1*) were detected with RT-qPCR. The primers of target genes are listed in Table S1.

### 2.13. Measurement of Intracellular Acetyl-CoA

Two groups of knockout cells and two groups of wild cells were cultured at the same time, with three replicates in each group. When the cells reached 70% confluence, one group of knockout cells and one group of wild cells were randomly treated with 100 μM acetyl-CoA salt (32140-51-5, Sigma, St. Louis, MO, USA, ≥93%) addition. After 48 h, the cells were collected, acetyl-CoA in the cells was extracted by using an acetyl-coenzyme assay kit (MAK039, Sigma, St. Louis, MO, USA) according to the manufacturer’s instructions. Then, the fluorescence intensity was measured (λ_ex_ = 535/λ_em_ = 587 nm) by a Biotek Microplate Reader (Winooski, VT, USA).

### 2.14. Measurement of Fatty Acid Metabolism Genes with Acetyl-CoA Addition

Acetyl-CoA salt (32140-51-5, Sigma, St. Louis, MO, USA, ≥93%) was added according to the above method, and the cells were collected. The mRNA expression levels of fatty acid synthesis genes (*ACC, FASN*), triacylglycerol synthesis genes (*DGAT1, DGAT2, AGPAT6*), lipid droplet formation and secretion genes (*PLIN2, TIP47*), and fatty acid regulatory factor genes (*SREBP1, LXRα*) were detected by using *UXT* and *GAPDH* as internal reference genes. The primers of target genes are listed in Table S1.

### 2.15. Statistical Analysis

The MAT-KO (*n* = 3) group and the NC group (*n* = 3) were taken as the experimental subjects, and the experimental data are shown as mean ± *SEM*. *T*-test analysis was performed on the data of the two groups using SPSS 19.0 statistical software (SPSS, Inc., Chicago, IL, USA), the difference was significant when *p* < 0.05 and extremely significant when *p* < 0.01.

## 3. Results

### 3.1. Evaluation of sgRNA for CRISPR-Mediated Repression of MAT

**Figure 1:**
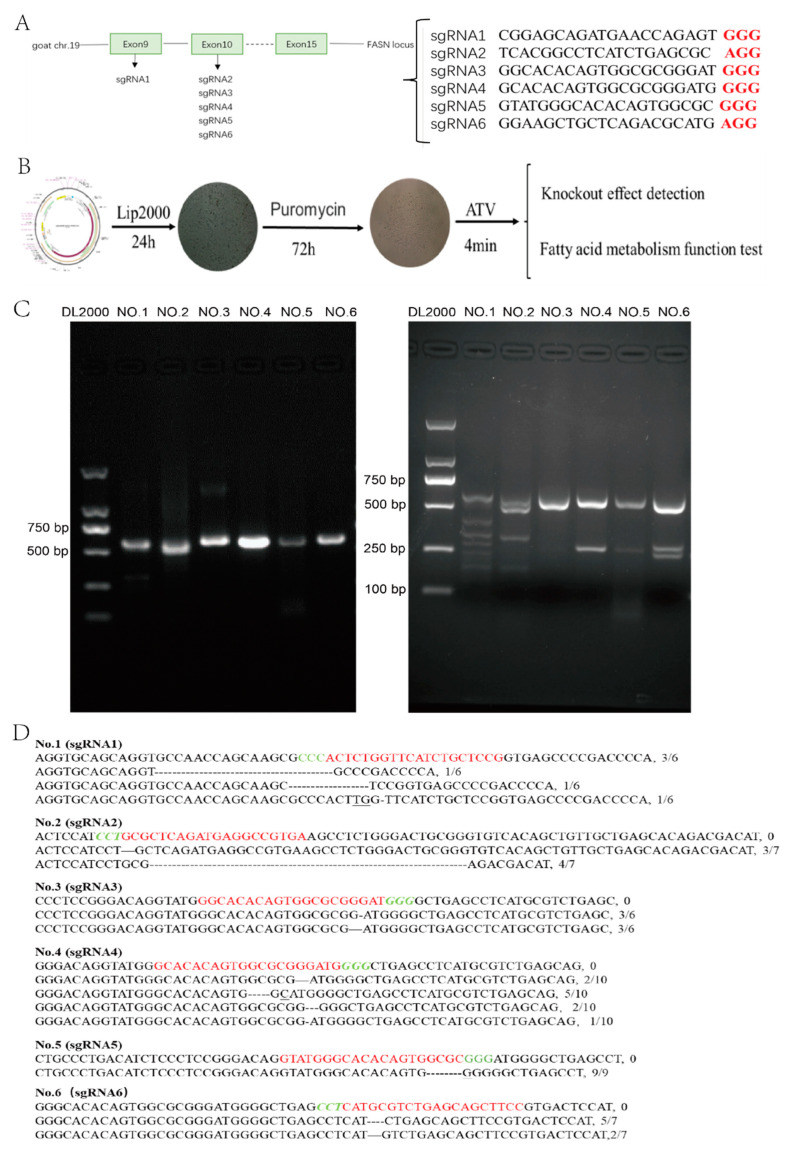
sgRNA design and screening. (**A**) Schematic illustration of the sites and sequence of sgRNA. Red is the PAM sequence. (**B**) Outline of the experimental protocol. Lip2000—transfection reagents; Puro—puromycin; ATV—cell dissociation solution. (**C**) Target fragment amplification and cutting efficiency. DL2000—DNA marker; NO.1—cells knockout by sgRNA 1; and others in the same way. NO.1 = 168 bp + 419 bp, NO.2 = 361 bp + 226 bp, NO.3 = 296 bp + 291 bp, NO.4 = 297 bp + 290 bp, NO.5 = 291bp + 296 bp, NO.6 = 328 bp + 259 bp. (**D**) Genotypes of sgRNA knockout. The first line was a wild-type sequence, with red indicating a sgRNA sequence and green indicating a PAM sequence, and the proportion of detected genotypes was displayed on the right side.

SgRNA vectors were successfully constructed and transfected into GMECs (Figure 1A,B). Thirty-one independent cells groups were obtained by puromycin screening. The efficiency of each sgRNA was detected by T7EN1 digestion (Figure 1C) and sequencing (Figure 1D), all six sgRNAs had gene editing efficiency.

### 3.2. Decreased Expression of MAT in Genome-Modified GMECs

**Figure 2:**
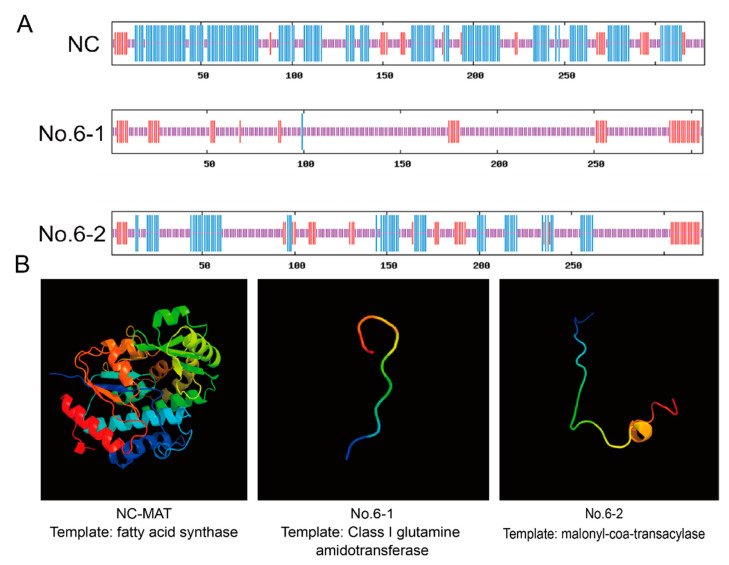
The bioinformatics analysis of MAT knockout efficiency. (**A**) Secondary structure analysis. NC—negative control cells; NO.6-1—a genotype of NO.6; NO.6-2—another genotype of NO.6; long vertical line—*α*-helix; middle vertical line—*β*-fold; Short vertical bar—irregular curl. (**B**) Tertiary structure analysis. From N to C, it is red, orange, yellow, green, cyan, blue, and purple, respectively.

**Figure 3:**
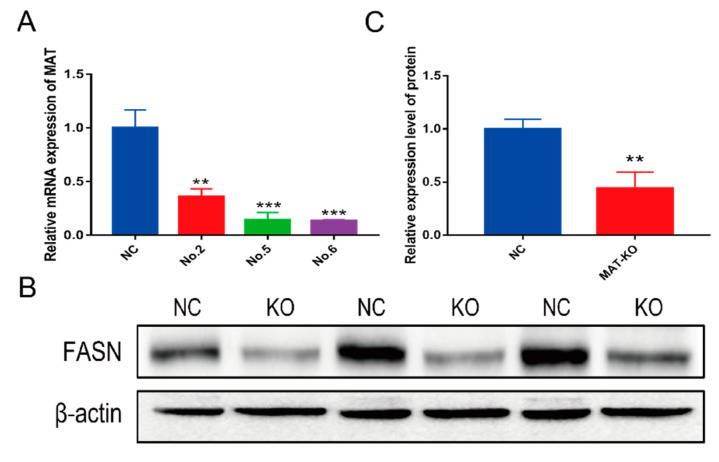
The trial analysis of MAT knockout efficiency. (**A**) MAT knockout efficiency at mRNA level. NC—negative control cells; NO.1—cells knockout by sgRNA 1; and others in the same way. (**B**) Protein changes of FASN in knockout cells. KO—MAT-knockout group (**C**); quantitative analysis of protein; MAT-KO—MAT-knockout group; ** *p* < 0.01, *** *p* < 0.001.

The screened monoclonal cells of No.2, No.5, and No.6 were resuscitated and tested. In the process of culturing, it was found that the No.2 cells showed obvious morphological changes; No.5 cells grew slowly, and No.6 cells were more normal. In the physicochemical properties prediction results, except for the knockout genotype of No 2-2, the physicochemical properties of the other knockout genotypes were changed significantly (Table S3). By structural prediction (No.6 cells, for example), it is found that the secondary structure (Figure 2A) and tertiary structure (Figure 2B) of the knockout type changed distinctly. The content of *α*-helix structure was obviously reduced. The mRNA expression levels of *MAT* gene in all three cells decreased significantly (*p* < 0.01) (Figure 3A). This included a 64.34% reduction in No 2 monoclonal cells, an 89.41% drop in No 5 monoclonal cells, and an 87.87% fall in No 6 monoclonal cells. FASN protein level decreased about 51.83% in No 6 monoclonal cells (Figure 3B,C). No 6 monoclonal cells could be used as an initial choice for the following experiment.

### 3.3. The Monoclonal Cells without Off-Target Effects

**Figure 4:**
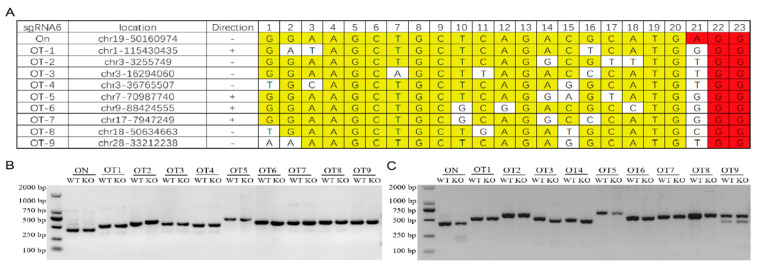
Off-target detection. (**A**) High-probability targeting site information of sgRNA 6 sequence in goat genome. “OT”—off-target site; the yellow highlight represents a coincident target sequence; the red highlight represents a coincident PAM sequence. (**B**) Target site amplification. “ON”—target site; “OT”—off-target site; “WT”—wild-type; “KO”—knockout. (**C**) Enzyme digestion detection of off-target site.

Ten target sites with high probability of sgRNA 6 in the genome were predicted (Figure 4A). By amplifying the target fragment containing each target site and T7EN1 enzyme digestion (Figure 4B), no detectable off-target effects were found at these sites (Figure 4C).

### 3.4. Knockout of MAT Suppresses Medium-Chain Fatty Acids and Triacylglycerol Synthesis

Knockout of *MAT* downregulated the relative contents of triacylglycerol and medium-chain fatty acids in cells (*p* < 0.05), upregulated the relative content of unsaturated fatty acids (*p* < 0.01). The percentages of C12:0 and C14:0 in total fatty acids were downregulated (*p* < 0.05), while C17:0, C18:2, and C18:1 were significantly increased (*p* < 0.05) (Table 1).

### 3.5. Knockout of MAT Decreased the Expression Levels of Genes Related to De Novo Fatty Acid Synthesis

**Figure 5:**
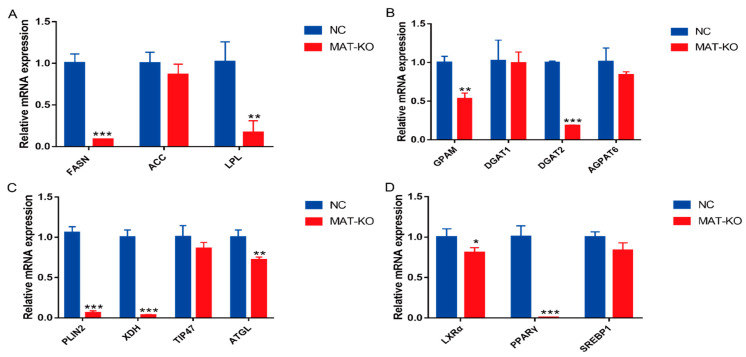
Effects of MAT-knockout on genes related to fatty acid metabolism. (**A**) Fatty acid synthesis and triacylglycerol hydrolysis. (**B**) Triacylglycerol synthesis (**C**) Lipid droplet formation, secretion and hydrolysis. (**D**) Regulatory factors. NC—negative control group; MAT-KO—knockout group; values are means ± *SEM* for three independent experiments. * *p* < 0.05, ** *p* < 0.01, *** *p* < 0.001.

Quantitative analysis showed that the expression level of *FASN* gene was significantly decreased (*p* < 0.001) (Figure 5A), this indicated that the knockout of key functional domains has a great influence on the expression of entire gene. The expression levels of triacylglycerol hydrolysis genes *LPL* and *ATGL*, triacylglycerol synthesis genes *GPAM* and *DGAT2,* lipid droplet formation and secretion genes *PLIN2* and *XDH,* and regulatory factors *LXRα* and *PPARγ* were all decreased (*p* < 0.05) (Figure 5). These results demonstrated that MAT elimination inhibited the anabolism of fatty acids.

### 3.6. Acetyl-CoA Suppresses the Fatty Acid Anabolism

**Figure 6:**
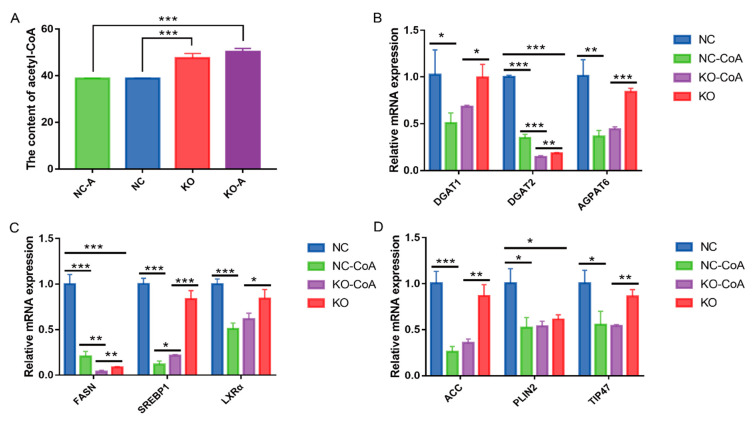
Acetyl-CoA suppresses the fatty acid anabolism. (**A**) Effect of MAT knockout on intracellular acetyl-CoA. NC-A—the control group added with acetyl-CoA; KO-A—the knockout group added with acetyl-CoA. (**B**–**D**) Effect of acetyl-CoA treatment on genes related to fatty acid synthesis. Values are means ± *SEM* for three independent experiments. * *p* < 0.05, ** *p* < 0.01, *** *p* < 0.001.

The content of acetyl-CoA in the knockout cells was significantly higher than that in the negative control group (*p* < 0.001) (Figure 6A). The intracellular content of acetyl-CoA was almost unchanged after treatment with acetyl-CoA in knockout cells (*p* > 0.05). Conversely, after treatment with acetyl-CoA, the expression levels of de novo fatty acid synthesis genes *ACC* and *FASN* (Figure 6C,D)*,* triacylglycerol synthesis genes *DGAT1, DGAT2*, and *AGPAT6* (Figure 6B), regulatory factors *SREBP1* and *LXRα* (Figure 6C), and lipid droplet formation genes *PLIN2* and *TIP47* (Figure 6D) were significantly downregulated (*p* < 0.05). These results suggested that FASN may be used to regulate fatty acid synthesis by affecting acetyl-CoA content.

## 4. Discussion

Fatty acid synthase is a key enzyme for endogenous fatty acid synthesis, and MAT is its functional domain [40]. MAT has both transferase and thioesterase activity in ruminants, which is involved in the initiation, extension, and termination of fatty acid synthesis. In this study, the deletion of MAT functional domain resulted in a 91.19% decrease in FASN mRNA and a 51.83% drop in FASN protein. This indicates that MAT plays a pivotal role in the expression of FASN, laying a foundation for the application of crucial functional domain knockout in large fragment genes.

The mechanism of FASN is different in ruminant and non-ruminant [27]. The average length of fatty acid chains produced in the mammary glands of ruminants is shorter than that produced in other tissues [47]. This may be related to the medium-chain thioesterase activity of FASN in ruminants [27]. MAT performs thioesterase activity and terminates fatty acid synthesis to produce short- and medium-chain fatty acids (<16C) in ruminants [7]. Interestingly, FASN gene expression is controlled mainly at the transcriptional level [7]. Knockout at the genomic level is easy to achieve good results. In this study, the deletion of FASN in GMECs caused the decrease in the medium-chain fatty acid contents. These results indicate that the FASN has a pivotal effect on the fatty acid composition of GMECs, which is expected to provide a basis for the directional change of milk composition.

Triacylglycerol is a nutrient-dense component of milk, and fatty acids are the valid basis for the synthesis of triacylglycerol [48]. The first step in triacylglycerol synthesis is catalyzed by GPAM, which is the rate-limiting enzyme for triacylglycerol synthesis [49]. AGPAT catalyzes the acylation of lysophosphatidic acid, among them, AGPAT2 plays a major role in adipose tissues and AGPAT6 functions predominantly in mammary tissues [50]. DGATs are a function-specific protein in the synthesis of triacylglycerol, and it catalyzes the third step in the synthesis of triacylglycerol. DGATs are divided into DGAT1 and DGAT2, which act on exogenous fatty acids and endogenous fatty acids, respectively [51]. The formation of lipid droplets is controlled by TIP47, XDH, and ADFP [29]. Secretion is accomplished by cooperation of BTN1A1, ADFP, and XDH [52,53]. PLIN2 maintains the stability of triacylglycerol, prevents their hydrolysis by esterases, and promotes the formation of cytoplasmic lipid droplets in GMECs [54]. ATGL, HSL, and LPL play crucial roles in the metabolism of triacylglycerol into free fatty acids [55]. Regulatory factors such as PPAR, LXR, and SREBP1 [56] affect milk fat content by regulating the expression of fatty acid synthesis genes, and the regulatory role of LXR is dependent on SREBP1 [57]. In this study, MAT knockout inhibited the expression of the above genes, slowed down the whole process of triacylglycerol synthesis, and finally reduced the triacylglycerol content (*p* < 0.05), indicating that MAT functional domain plays a pivotal role in triacylglycerol synthesis.

Acetyl-CoA is a powerful intracellular metabolic intermediate that determines the balance of catabolism and anabolism in lipid metabolism [58]. Normally, mitochondrial acetyl-CoA is taken as a relatively independent part, and acetyl-CoA in cytoplasm and nucleus is taken as a whole [59]. Acetyl-CoA in mitochondria is produced by *β*-oxidation of fatty acids. Acetyl-CoA in cytoplasm is converted from acetic acid or produced by decomposition of citric acid in mitochondria with ATP-citrate lyase [60]. Acetyl-CoA in the nucleus is converted by citric acid or pyruvic acid and interacts with the acetyl-CoA content in the cytoplasm. Acetyl-CoA in cytoplasm forms malonyl-CoA under the catalysis of ACC, then forms fatty acid under the catalysis of FASN. In the nucleus, acetyl-CoA regulates gene expression by affecting the acetylation of transcription factors such as PPARγ and histone acetylation [61,62]. Among them, it is established in ruminants that PPARs are capable of utilizing milk fatty acids as ligands and modulate the expression of genes involved in lipid metabolism [63]. In this study, the treatment of acetyl-CoA in cells significantly reduced the expression of genes related to fatty acid synthesis, and *MAT* knockout accumulated acetyl-CoA content in cells, indicating that FASN regulates fatty acid synthesis by regulating the content of acetyl-CoA.

The limitation of this work is that our study only explored the knockout of FASN in GMECs. In particular, total FASN knockout in mice is fatal to embryos, and even heterozygous mice show impaired growth and survival [64]. Mammary specific FASN deletion hinders breast development and induces premature breast degeneration [31]. Therefore, the toxic effect of FASN knockout on tissue must be considered, which is likely to affect the observed phenotype.

## 5. Conclusions

FASN is a core enzyme in de novo synthesis of fatty acids, and MAT is a pivotal functional domain of FASN. Moreover, FASN plays a crucial role in the synthesis of medium-chain fatty acids in ruminant, which may be related to the MAT functional domain’s transferase and thioesterase activities. This study proves that MAT knockout in GMECs leads to a decrease in medium-chain fatty acids and triacylglycerol. Our findings provide the basis for altering the composition of milk fatty acids by gene editing.

## Figures and Tables

**Table 1:** Effects of MAT-knockout on fatty acid composition and triacylglycerol content in GMECs.

Fatty Acid	NC	MAT-KO	Fatty Acid	NC	MAT-KO
C10:0 (%)	2.73 ± 0.44	1.56 ± 0.12	C18:1 (%)	10.09 ± 0.40	11.75 ± 0.17 *
C12:0 (%)	3.05 ± 0.28	1.81 ± 0.04 *	C18:0 (%)	24.18 ± 0.99	24.94 ± 0.12
C14:0 (%)	10.40 ± 0.47	8.48 ± 0.07 *	C20:4 (%)	0.21 ± 0.02	0.29 ± 0.02
C15:0 (%)	0.88 ± 0.04	0.90 ± 0.02	C22:6 (%)	0.10 ± 0.02	0.10 ± 0.02
C16:1 (%)	0.24 ± 0.03	0.32 ± 0.00	MCFA (%)	5.78 ± 0.68	3.37 ± 0.13 *
C16:0 (%)	46.19 ± 1.23	47.30 ± 0.18	PUFA (%)	1.13 ± 0.10	1.67 ± 0.82 *
C17:1 (%)	0.15 ± 0.02	0.20 ± 0.02	UFA (%)	11.61 ± 0.44	13.93 ± 0.21 **
C17:0 (%)	0.96 ± 0.02	1.08 ± 0.02 *	SFA/UFA	7.64 ± 0.32	6.18 ± 0.11 *
C18:2 (%)	0.82 ± 0.07	1.28 ± 0.05 **	TAG (mg/g protein)	285.64 ± 23.30	182.97 ± 12.12 *

Fatty acid data are shown as ratio of the total fatty acids (%). Statistical analysis was performed with Student’s *t* test. MCFA—medium-chain fatty acid; PUFA—polyunsaturated fatty acid; UFA—unsaturated fatty acid; TAG—triacylglycerol. Values are presented as means ± *SEM*. * *p* < 0.05, ** *p* < 0.01.

## Data Availability

Data is contained within the article or supplementary material.

## Supplementary Materials

The following supporting information can be downloaded at: https://www.mdpi.com/article/10.3390/foods11091291/s1, Table S1: Characteristics of Primers Used in the RT-qPCR Reaction; Table S2: Amplification primer of sgRNA 6 target site sequence; Table S3: Physicochemical properties of knockout genotypes.

## Abbreviations

ACC	acetyl-coenzyme A carboxylase alpha
AGPAT6	1-acylglycerol-3-phosphate O-acyltransferase 6
ATGL	adipose triacylglycerol lipase
DGAT1	diacylglycerol acyl transferase 1
DGAT2	diacylglycerol acyl transferase 2
FASN	fatty acid synthase
GAPDH	glyceraldehyde-3-phosphate dehydrogenase
GPAM	glycerol-3-phosphate acyltransferase 1
LPL	lipoprotein lipase
LXRα	liver X receptors alpha
MAT	malonyl/acetyltransferase
PPARγ	Peroxisome-proliferator-activated receptor gamma
SREBP1	sterol regulatory element-binding transcription protein 1
TIP47	tail-interacting protein 47
UXT	ubiquitously expressed transcript
XDH	Xanthine dehydrogenase
