# Supplementary material for: Malonyl/Acetyltransferase (MAT) Knockout Decreases Triacylglycerol and Medium-Chain Fatty Acid Contents in Goat Mammary Epithelial Cells
Source: Foods. 2022 Apr 29;11(9):1291. doi: 10.3390/foods11091291 (PMC9104349; doi:10.3390/foods11091291)
Supplement: Supplementary file 1 [file foods-11-01291-s001.zip › foods-1664392-SM.pdf]

Supplemental Table S1. Characteristics of Primers Used in the RT-qPCR Reaction

Gene name	Sense Primer (5′–3′)	Anti-Sense Primer (5′–3′)	size (bp)
ACC	F: CTCCAACCTCAACCACTACGG	R: GGGGAATCACAGAAGCAGCC	171
AGPAT6	F: AAGCAAGTTGCCCATCCTCA	R: AAACTGTGGCTCCAATTTCGA	101
ATGL	F: GGAGCTTATCCAGGCCAATG	R: TGCGGGCAGATGTCACTCT	180
DGAT1	F: CCACTGGGACCTGAGGTGTC	R: GCATCACCACACACCAATTCA	101
DGAT2	F:CATGTACACATTCTGCACCGATT	R: TGACCTCCTGCCACCTTTCT	100
FASN	F: GGGCTCCACCACCGTGTTCCA	R: GCTCTGCTGGGCCTGCAGCTG	226
GAPDH	F: GCAAGTTCCACGGCACAG	R: GGTTCACGCCCATCACAA	249
GPAM	F: ATTGACCCTTGGCACGATAG	R: AACAGCACCTTCCCACAAAG	188
LXRα	F: CATCAACCCCATCTTCGAGTT	R: CAGGGCCTCCACATATGTGT	163
MAT	F: ATGGCGGCTGTAGGCTTGAC	R: GCACCTCCTTGGCGAACACG	169
PPARg	F: CCTTCACCACCGTTGACTTCT	R:GATACAGGCTCCACTTTGATTGC	145
SCD1	F : CCATCGCCTGTGGAGTCAC	R:GTCGGATAAATCTAGCGTAGCA	256
SREBP1a	F: CTGCTGACCGACATAGAAGACAT	R: GTAGGGCGGGTCAAACAGG	81
TIP47	F: GGTGGAGGGTCAGGAGAAA	R: TCACGGAACATGGCGAGT	123
UXT	F: TGTGGCCCTTGGATATGGTT	R: GGTTGTCGCTGAGCTCTGTG	101
XDH	F: GATCATCCACTTTTCTGCCAATG	R: CCTCGTCTTGGTGCTTCCAA	100

Supplemental Table S2. Amplification primer of sgRNA6 target site sequence

target sites	The first primer	length bp	The second primer	length bp
ON	F: CAGCAGCCATGCCCTTC	587	F: AGGTGCCAACCAGCAAGCG	415
	R: CGTCGGCATAGGCACAGG		R: TGGGAACAGGATGGGTCAGG	
OT1	F: TGTAGTCCATCAGGCTCTT	661	F: AGTTGCCATTTCTTCCT	513
	R: GCCCTGCCCAATAAAG		R: AGAATCCCAGCAGCAT	
OT2	F: GTCCGTTCACCTCCCAAGTA	645	F: GACCCAGAAGTGGTCATCCC	576
	R: ATCCCACCTCCAGCACCTA		R: CCCACCTCCAGCACCTACA	
OT3	F: GGGAGTAAACAACCGAAGAG	696	F: TTATTCGGAGCAGCAGTTT	421
	R: GGGTAGCGATGCCTGTG		R: AGCGGAACCTGGCACA	
OT4	F: GGAGCCAGTTGTGAATG	649	F: GTGAATGGGATTTGTTTG	491
	R: GGAGACAGCCGTGAAG		R: TGGACATTGCCTGAAGT	
OT5	F: GGAGGGCAAGATAGGC	633	F: GGGAAAGGAGACCATAAA	497
	R: AGACCAGCAACCCAAGA		R: AGACCAGCAACCCAAGA	
OT6	F: CCGGTGGACAGGGAAAGGA	661	F: GGTGGACAGGGAAAGGAGAC	519
	R: TAGGAGGCGGGACTGTGGAG		R: GCTGACTGATGCGTGGGTAT	
OT7	F: CTCGCTTCTGCGTCTTCA	717	F: GCTGCGGGATGTTGTGC	532
	R:GCCATCACATTACCTATCCCT		R: GCCTATGTTGGGTGCTGA	
OT8	F: GGGCAAGGCACGAGTGAGGT	648	F:CACGAGTGAGGTCCTCTTTCCC	554
	R:GCTGGGTGAGAACAGGAGCAG		R: GCATGATGTTCTCGGGCTTG	
OT9	F: GGGCAGCACTCATCAC	662	F: CTCTGGAAGCGAAGTGC	539
	R: AACCAGGTCCTAACCAAC		R: AACCAGGTCCTAACCAACT	

Supplemental Table S3. Physicochemical properties of knockout genotypes.

	WT	No.2-1	No.2-2	No.5	No.6-1	No.6-2
Number of bases missing	-0	-2	-57	-8	-4	-2
Theoretical value of PI	5.83	11.13	5.82	10.59	11.77	10.93
Coefficient of instability	53.35	58.25	53.96	59.73	98.53	60.51
Coefficient of fat	102.02	67.98	99.45	65.89	41.93	67.29
Total mean hydrophilicity	0.106	-0.691	0.088	-0.745	-0.483	-0.708
